# Genome-Wide Association Implicates Candidate Genes Conferring Resistance to Maize Rough Dwarf Disease in Maize

**DOI:** 10.1371/journal.pone.0142001

**Published:** 2015-11-03

**Authors:** Gengshen Chen, Xiaoming Wang, Junjie Hao, Jianbing Yan, Junqiang Ding

**Affiliations:** 1 National Key Laboratory of Crop Genetic Improvement, Huazhong Agricultural University, Wuhan, 430070, China; 2 Institute of Crop Science, Chinese Academy of Agricultural Sciences, Beijing, 100081, China; 3 Institute of Plant Protection, Henan Academy of Agricultural Sciences, Zhengzhou, 450002, China; 4 College of Agronomy, Synergetic Innovation Center of Henan Grain Crops and National Key Laboratory of Wheat and Maize Crop Science, Henan Agricultural University, Zhengzhou, 450002, China; International Rice Research Institute, PHILIPPINES

## Abstract

Maize rough dwarf disease (MRDD) is a destructive viral disease in China, which results in 20–30% of the maize yield losses in affected areas and even as high as 100% in severely infected fields. Understanding the genetic basis of resistance will provide important insights for maize breeding program. In this study, a diverse maize population comprising of 527 inbred lines was evaluated in four environments and a genome-wide association study (GWAS) was undertaken with over 556000 SNP markers. Fifteen candidate genes associated with MRDD resistance were identified, including ten genes with annotated protein encoding functions. The homologous of nine candidate genes were predicted to relate to plant defense in different species based on published results. Significant correlation (R^2^ = 0.79) between the MRDD severity and the number of resistance alleles was observed. Consequently, we have broadened the resistant germplasm to MRDD and identified a number of resistance alleles by GWAS. The results in present study also imply the candidate genes in defense pathway play an important role in resistance to MRDD in maize.

## Introduction

In maize, at least ten viruses caused significant agronomic losses globally [1]. Maize rough dwarf disease (MRDD) is one of the destructive viral diseases that result in direct yield loss in maize world [2–5]. It was firstly reported in 1954 in China, and became more and more popular since 1990s partly because of the changes in the cultivation patterns along with the repeated occurrence of warmer winters [6–7]. In recently years, there have been several serious disease outbreaks, especially in Huang-Huai-Hai region, a major summer corn belt in China. Yield loss caused by MRDD was estimated to be 20–30%, even as high as 100% in severely infected fields [8–9].

The typical symptoms of MRDD included severe dwarfing and stunting, dark-green and leathery leaves, and at adult stage, the tassels and ears of diseased plants were malformed and underdeveloped. Three virus species in the genus *Fijivirus*, rice black streaked dwarf virus (RBSDV), maize rough dwarf virus (MRDV) and Mal de Rio Cuarto virus (MRCV), caused disease problems in East Asia, Europe and South America, respectively [10]. In China, the causal agent was initially designated as MRDV and later it was shown and confirmed to be RBSDV by sequencing virus isolates from maize in different regions of China [11].

Rice black streaked dwarf virus is transmitted by small brown planthopper (*Laodelphax striatellus*) in a persistent manner [12]. The outbreak of MRDD generally coincides with a high density of viruliferous planthopper during the most susceptible stage in maize [13]. Therefore, measures for controlling MRDD have been suggested, which include reducing vector populations through insecticide applications, delaying the sowing date to avoid high vector populations and improving field management [14]. These methods can in part alleviate the MRDD severity, but always with high risk and poor efficiency.

To control diseases in crops, application of host resistance is the most cost-effective and environmentally friendly approach [15]. In light of the severity of MRDD in China, numerous studies focused on screening resistant germplasm under natural-infection conditions [16–19]. However, limited number of highly resistant lines were identified, and the major source of resistance was derived from US hybrid P78599 [20–21].

Resistance to MRDD is quantitatively inherited and both major and minor QTL have been identified. Using the F_2:3_ derived-lines from a cross between Mo17 (susceptible) and BLS14 (resistant), Di et al. [22] detected two QTL for resistance to MRCV, which located on chromosome bins 1.03 and 8.03/4, respectively. A very recent research reported four QTL for resistance to MRCV, which located on chromosomes 1, 6, 8 and 10, respectively [23]. More QTL mapping studies on the resistance to MRDD were conducted in China. In a F_2_ population derived from a cross between inbred line 90110 (resistant) and line Ye478 (susceptible), at least three QTL were found on chromosome bins 6.02, 7.02 and 8.07, respectively [24–25]. In an RIL (recombinant inbred lines) population developed from a cross between X178 (resistant) and B73 (susceptible), a major QTL was identified for resistance to RBSDV on chromosome 8 (bin 8.03) [9], in which region one major QTL conferring recessive resistance to MRDD, *qMrdd1*, was also detected and fine-mapped into a region of 1.2 Mb in an independent experiment [26].

Although the availability of QTL has contributed substantially to our current understanding of the genetic basis of resistance to MRDD, QTL have been determined in linkage populations. In addition, the majority of the resistant sources selected for linkage-based QTL mapping studies in China were derived from the same US hybrid P78599 [27]. Consequently, the QTL from resistant lines with narrow genetic basis is subject to low allele numbers and may represent only a portion of the genetic control of resistance to MRDD in maize.

In contrast to linkage mapping, genome-wide association study (GWAS) is an alternative method for detecting complex genetic traits in plants [28–29]. GWAS takes full advantage of the natural variations within germplasm collection to identify the genetic loci underlying traits at a relatively high resolution. In recent years, rapid development in next generation sequencing and genotyping technologies is a major driving force for GWAS [30]. GWAS are now routinely applied to detect the genetic architecture as well as identify causative factors for agronomic traits in plants [31]. In the last years, GWAS has been proven powerful in revealing the complex genetic basis of many phenotypes in crop plants such as maize, rice, wheat, barley and other 17 plant species [32]. In maize, encouraging GWAS results have been reported for flowering time, plant architecture, disease resistance, kernel composition and secondary metabolite concentrations [33].

As an outcrossing species, maize is an ideal crop for association mapping for its abundant genetic diversity and rapid linkage disequilibrium (LD) decay. Therefore, the power of association studies can be significantly improved by increasing the number of individuals of the experimental population, precise phenotyping and genotyping with high-density SNPs [34–35]. In present study, considering the narrow genetic basis of resistance to MRDD in Chinese maize germplasm, a global collection of 527 diverse lines representing the major temperate and tropical/subtropical breeding programs of China, Germplasm Enhancement of Maize (GEM) and CIMMYT were evaluated for resistance to MRDD in multiple environments, and GWAS were performed with over 556000 high-throughput SNPs to determine the genetic architecture as well as causative genes for resistance to MRDD in maize.

## Materials and Methods

### Experimental design and Phenotype evaluation

A global collection of 527 diverse lines with temperate, tropical and subtropical origin was used for association mapping. This collection included 238 lines from maize breeding programs in China, 54 lines from the Germplasm Enhancement of Maize (GEM) project and 235 lines from the CIMMYT maize breeding programs. All the lines have been well described in previous studies [36] and detailed information also can be downloaded at http://www.maizego.org/resource.html.

For the evaluation of resistance to MRDD, field trials were conducted in three locations with four environments under epiphytotic of RBSDV: Jiangsu (Yancheng, N33°22′, E120°08′) in 2011, Shandong (Jining, N35°23′, E116°35′) in 2011 and 2012, Henan (Kaifeng, N34°47', E114°20′) in 2013. At the three experimental locations, the institute of crop science belonging to the Chinese Academy of Agricultural Sciences has set up experimental field bases for non-profit agricultural research with a wide array of partners in China. In present study, the field experiments of the natural occurrence of MRDD in all the three experimental stations were approved by the institute of crop science. Further, the experimental stations where field studies were conducted are not protected locations for endangered or protected species. The lines in association panel were divided into two groups (temperate and tropical/subtropical) based on pedigree information, and incompletely randomized block design was used with two replications per location. Each plot consisted of single row of 0.7 m in width and 3.0 m in length. To make viral inoculation more likely, planting date in each year and location may fluctuate between May 10 and May 20 to coincide with planthopper infestation. In each location, inbred lines Qi319 and Zheng58 were planted in each block as a resistant and susceptible check, respectively. Qi319 was developed from US hybrid P78599 which was the major source carrying resistance to MRDD in Chinese maize breeding program [20–21], and Zheng58 was one of the parental lines of an elite hybrid Zhengdan 958 widely planted in China in recent years. Maize resistance to RBSDV was evaluated using the (1–5) rating scale during the maturity stage [37]. The rating “1” and “5” indicated the most resistant phenotype and the most susceptible phenotype, respectively. Then the disease severity index (DSI) was used to represent MRDD severity of each line in the association panel, which was calculated based on disease severity rating: DSI (%) = ∑ (rating × number of plants in rating) × 100 / (5 × total number of plants) [38].

### Statistical analyses

The phenotypic data collected from multiple environments were subjected to the following methods for analyzing different parameters. Analysis of variance was performed using SAS 9.1 program. Components of variance were estimated using a complete random effects model, and broad-sense heritability was calculated considering the percentages of genotypic variance, over the total phenotypic variance including genotype by environment variance and error variance components [39]. To minimize the effect of environmental variation, phenotypic BLUPs (best linear unbiased predictions) were used for association studies.

### Genotyping

The lines of the association panel were genotyped by two genotyping platforms, MaizeSNP50 BeadChip and the Sequenom MassArray iPLEX. In total 1.03 million high-quality SNPs genotyped by RNA-seq and 56110 SNPs genotyped by the MaizeSNP50 BeadChip were achieved. In this study, 556809 SNPs with a moderate minor allele frequency (MAF>5%) were employed in the association analysis. The detailed information was described in recent studies [40–41], and can be downloaded at http://www.maizego.org/resource.html.

### Genome-wide association analysis

GWAS was performed by using a mixed linear model (MLM) approach [42–43]. Both population structure (Q) and kinship (K) were taken into account during the GWAS with MLM to avoid spurious associations. The detailed information of Q and K of the association panel was described in previous study [36]. Briefly, the population structure and kinship information were input using the softwares STRUCTURE [44] and SPAGeDi [45], respectively. In the STRUCTURE analysis, *K* = 3 was considered as the best possible numbers of subpopulations, which was consistent with the known pedigree and germplasm of the association panel. *P* value of each SNP was calculated and significance was defined at a uniform threshold of *P*≤1.79×10^−6^ (*P* = 1/n; n = total markers used, which was roughly a Bonferroni correction). SNP with the lowest *P* value was reported for each significant locus, and corresponding gene was predicted from the annotated maize genome based on maize inbred B73 reference genome (http://www.maizegdb.org/gbrowse).

## Results

### Phenotypic variations for MRDD resistance in association panel

The field trial was conducted in four environments (i.e., Yancheng and Jining in 2011, Jining in 2012 and Kaifeng in 2013) to evaluate the resistance to MRDD. We first evaluated the reactions of resistance to MRDD in resistant check (Qi319) and susceptible check (Zheng58) in each environment. In 2011, due to the outbreak of MRDD, Qi319 was highly susceptible in each block of Yancheng and Jining locations (i.e., the mean DSI were 73.3 and 100 in Yancheng and Jining, respectively). Consequently, no significant variation was observed between Qi319 and Zheng58. However, we screened 11 and 5 lines extremely resistant to MRDD in Yancheng and Jining, respectively. In 2012 and 2013, significant difference was observed between Qi319 and Zheng58, where Qi319 was highly resistant while Zheng58 was highly susceptible to MRDD. Large phenotypic variations in association panel were observed for MRDD severity in 2012 and 2013. Therefore, the phenotypic data from 2012 and 2013 together with the extremely resistant lines (11 lines from Yancheng and 5 lines from Jining) in 2011 were included for further analysis.

Significant variance components for genotype (G) and genotype × environment (G×E) interactions were observed in the combined analysis. However, G×E interactions represented only a small fraction of the total variance. Heritability estimates for MRDD resistance across the environments was 0.80, which meant that much of the phenotypic variation was derived from genetic factors and suitable for further association mapping. Entry-based means, ranges, variation components and heritability over all environments for MRDD resistance were presented in Table 1, and the phenotypic variation of resistance to MRDD across the environments was shown in S1 Fig and S1 Table.

**Table 1:** Analysis of variance, heritability for resistance to MRDD in 527 inbred lines.

Parameter	Disease severity index
mean(Mi)	89.2
range(Mi)	23–100
*σ* ^2^_*g*_	58.9
*σ* ^2^_*ge*_	14.7
*h* ^*2*^	0.80

Mi is the adjusted entry mean of genotype *i* calculated based on the phenotypic data in 2012 (Jining), 2013 (Kaifeng) and highly resistant lines in 2011 (i.e., 11 lines from Yancheng and 5 lines from Jining).

*σ* ^2^_*g*_ and *σ* ^2^_*ge*_ are the genotype and genotype × environment interaction variances.

*h* ^2^ is the heritability on an entry-mean basis.

### Association analysis

**Fig 1:**
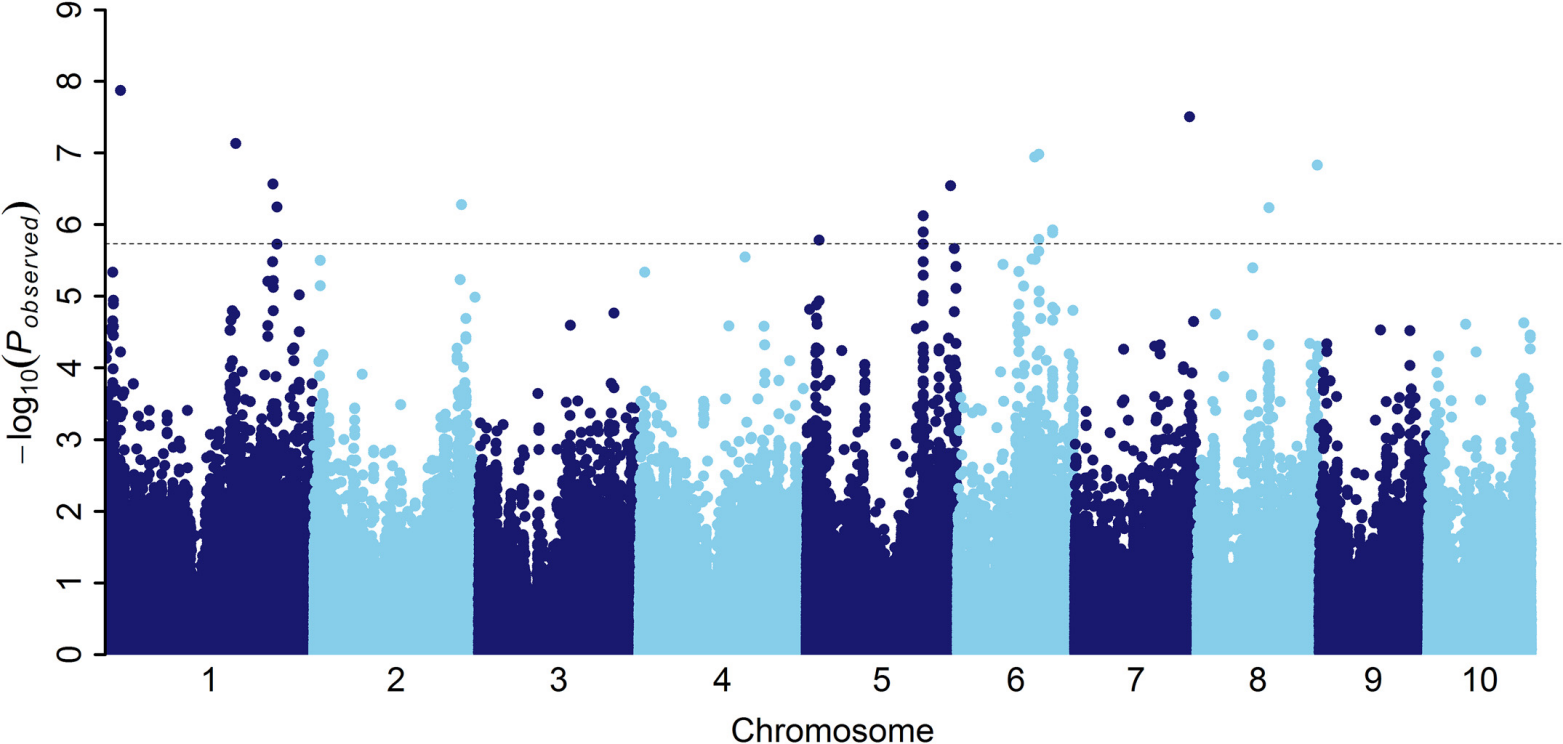
Manhattan plot resulting from the GWAS results for disease severity index of MRDD. The dashed horizontal line depicts the Bonferroni-adjusted significance threshold (*P* = 1.79×10^−6^).

**Fig 2:**
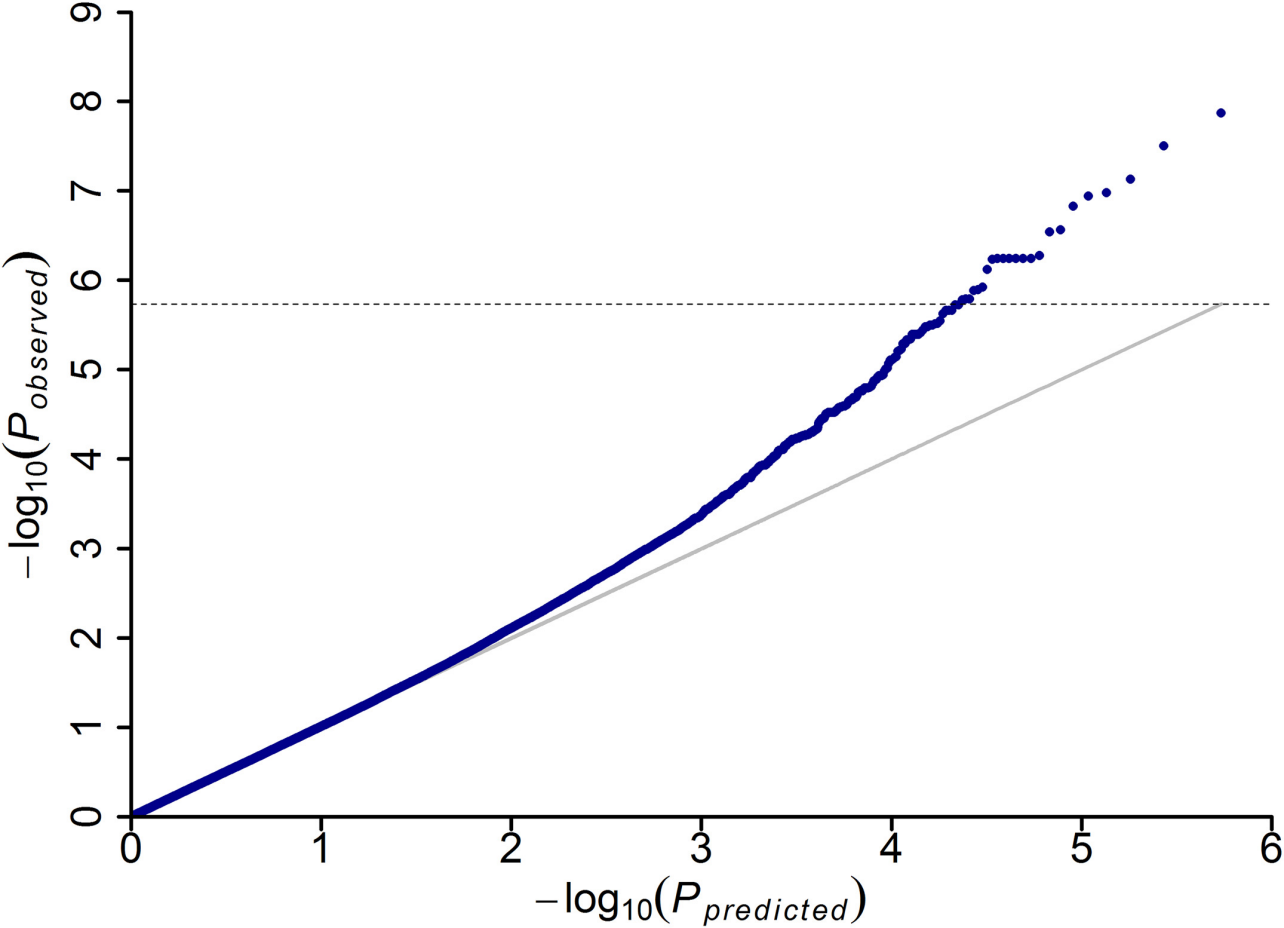
Quantile-quantile plot resulting from the GWAS results for disease severity index of MRDD.

To minimize the effect of environmental variation, phenotypic BLUPs across environments (i.e., the phenotypic data from 2012 and 2013 together with the extremely resistant lines in 2011) were used for association studies. To detect the genotypic variation underlying the resistance to MRDD, the significance of association between DSI and the genome-wide 556809 SNPs with MAF≥0.05 was evaluated by MLM analysis using kinship relationship (K matrix) and population structure (Q matrix) as covariate. The manhattan plot indicated that a total of 17 loci reached the genome-wide significance threshold of *P*≤1.79×10^−6^ (Table 2 and Fig 1). The quantile-quantile (QQ) plots were generated to detect inflation of statistics due to population stratification (Fig 2). The numbers of significant loci varied from chromosome to chromosome, and the maximum number of significant loci was observed on chromosomes 1 and 6, on which five each significant loci were detected, followed by three on chromosome 5, two on chromosome 8 and one each on chromosomes 2 and 7. For all the 17 loci significantly associated with MRDD DSI, five resistance alleles had relatively low frequences than their counterparts, and phenotypic effect explained by each allele varied from 5.4% to 7.8% in the association panel.

**Table 2:** Candidate genes, chromosomal position and SNPs significantly associated with resistance to MRDD.

Chr. bin	SNPs	Allele^a^	MAF^b^	*P* value	R^*2*^	Candidate genes^c^	Annotation^d^
1.02	chr1.S_22502510	G/A	0.10	1.35E-08	0.078	*GRMZM2G457178*	Uncharacterized protein
1.06	chr1.S_189085299	T/G	0.07	7.40E-08	0.067	*GRMZM2G055992*	Antifreeze protein
1.08	PZE-101195153	C/A	0.08	2.73E-07	0.062	Intergenic	
1.08	chr1.S_248461992	G/A	0.05	5.71E-07	0.059	*AC196066*.*3_FG003*	Antifreeze protein
1.08	chr1.S_248515591	G/A	0.05	5.69E-07	0.059	*GRMZM2G417089*	Lysine-specific demethylase
2.08	chr2.S_213342002	T/G	0.06	5.31E-07	0.061	*GRMZM2G092877*	Uncharacterized protein
5.03	chr5.S_18700960	C/A	0.10	1.65E-06	0.060	*GRMZM2G152764*	Uncharacterized protein
5.04	chr5.S_168672030	A/C	0.08	7.60E-07	0.056	*GRMZM2G113332*	Antifreeze protein
5.07	chr5.S_208163750	T/C	0.35	2.88E-07	0.066	*GRMZM2G055204*	Ethylene-responsive transcription factor
6.04	chr6.S_112004260	G/A	0.06	1.14E-07	0.066	*GRMZM2G073700*	RNA-binding protein
6.04	chr6.S_117803725	G/A	0.07	1.05E-07	0.070	*GRMZM2G132373*	phosphatidylinositol kinase
6.04	chr6.S_117823578	T/C	0.07	1.62E-06	0.057	*GRMZM2G431708*	Phosphogluconate dehydrogenase
6.05	chr6.S_137515197	T/C	0.10	1.20E-06	0.054	Intergenic	
6.05	chr6.S_137651201	C/T	0.09	1.31E-06	0.054	*GRMZM2G055699*	Beta-glucosidase
7.04	chr7.S_165848597	A/T	0.07	3.16E-08	0.072	*GRMZM2G048846*	Uncharacterized protein
8.03	PZE-108058210	A/G	0.20	5.81E-07	0.059	*GRMZM2G110739*	MLO-like protein
8.08	chr8.S_173507343	C/A	0.08	1.49E-07	0.068	*GRMZM2G437859*	Uncharacterized protein

^a^Major allele, minor allele; underlined bases are the resistance alleles.

^b^MAF stands for minor allele frequency.

^c^A plausible biological candidate gene in the locus to the lead SNP.

^d^Each candidate gene is annotated according to InterProScan.

### Candidate genes co-localized with associated SNPs

To understand the causes of variation in resistance to MRDD, we examined the candidate genes co-localizing with SNPs based on the publicly available B73 annotated genome (http://www.maizegdb.org/gbrowse). After excluding 2 of 17 SNPs within the inter-genic regions, we identified 15 candidate genes associated with resistance to MRDD, which included 10 genes of annotated protein encoding functions and 5 genes with uncharacterized protein. Remarkably, nine of the 10 protein encoding genes were predicted to relate to plant defense (Table 2), including three antifreeze proteins [46–48], a lysine-specific demethylase [49], an ethylene-responsive transcription factor [50–51], a phosphatidylinositol kinase [52], a phosphogluconate dehydrogenase [53], a beta-glucosidase [54] and a MLO-like protein [55–56].

### Combined analysis of the number of resistance alleles with MRDD resistance

**Fig 3:**
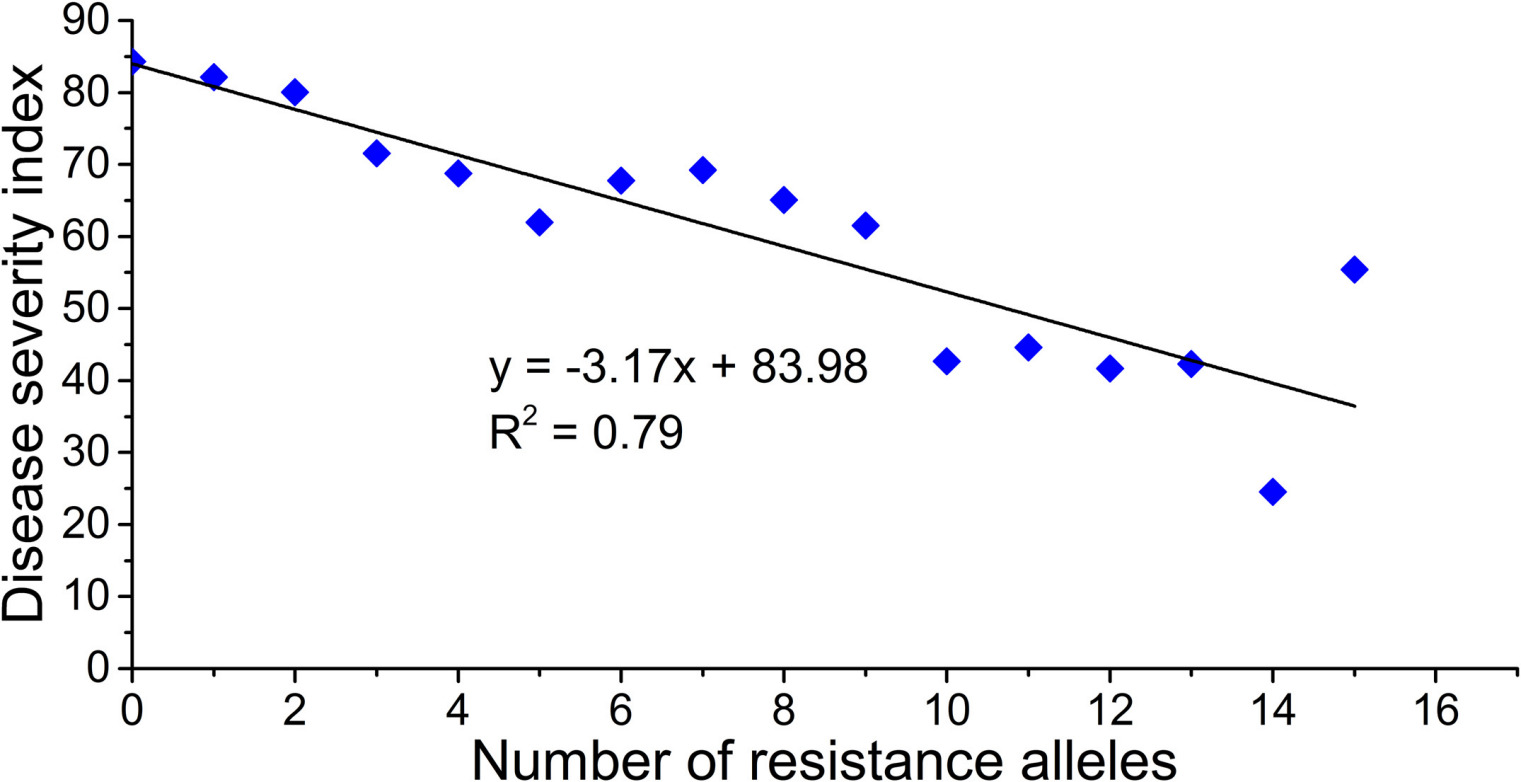
The joint effect of resistance alleles on resistance to MRDD in association panel.

To further understand the combined effect of causes of variation in resistance to MRDD, we examined the number of resistance alleles in each line in association panel. For the 17 loci significantly associated with resistance to MRDD, the resistance alleles of the lines ranged from 0 to 15. We regrouped the lines based on the number of resistance alleles they carried. The relationship between MRDD resistance and the number of resistance alleles was estimated with linear regression analysis. On group level, we found the number of resistance alleles was significantly associated with MRDD resistance. As shown in Fig 3, the number of resistance alleles increased and so did mean resistance to MRDD in the group. There was a strong and negative correlation between the MRDD severity and number of resistance alleles (R^2^ = 0.79). We also analyzed the correlation between MRDD severity of line in association panel and the number of resistance alleles in each line, and similar results were also achieved (S2 Fig).

## Discussion

In this study, we perform GWAS to detect the genetic architecture controlling natural variation in MRDD resistance in maize. The initial incentive of present GWAS is the severity of MRDD and the narrow genetic basis of resistant germplasm in maize breeding program in China. MRDD is extremely hard to control once the plants are infected. This has led to plant breeders referring the destructive viral disease as maize cancer. In light of the importance of MRDD in Chinese maize breeding program, numerous studies have been conducted to screen the resistant germplasm and determine the resistance genetic mechanism. However, the major source of resistance was derived from US hybrid P78599 [20–21], in which the major gene was mapped with varying resolution by linkage analysis [9, 26]. Recently, GWAS was also conducted in two studies, in which 236 and 184 Chinese maize inbreds were used for association analysis, respectively [27, 57]. The major advances here are: 1) we used large size of association mapping panel with high genetic diversity. Since limited number of highly resistant lines in Chinese maize germplasm, we enlarged the association mapping population to 527 diverse lines with the global collection, which included over 200 tropical/subtropical lines from CIMMYT maize germplasm known for resistance to multiple maize diseases; 2) we evaluated the resistance to MRDD in multiple environments; 3) we used high-density SNPs (556000 with MAF greater than 0.05) to perform GWAS. We comprehensively describe the genetic architecture and identify a set of specific genes implicated in controlling the resistance to MRDD in maize.

We used the publically available maize genome sequence to identify candidate genes encompassing the SNPs associated with resistance to MRDD. Remarkably, most of the genes identified were predicted to function in plant defense pathways (Table 2). There were 3 genes (*GRMZM2G055992*, *AC196066*.*3_FG003* and *GRMZM2G113332*) encoding antifreeze protein. This type of genes has high similarity to pathogenesis-related (PR) proteins and has been shown to enhance disease resistance [46–48]. One candidate gene (*GRMZM2G417089*) contains Jumonji C domain, known to encode a histone lysine demethylase. It was reported in rice that Jumonji C protein gene JMJ705 specifically reverses H3K27me2/3 and involved in defense-related gene activation [49]. One gene (*GRMZM2G055204*) encodes transcription factor involving in ethylene pathway, and can integrate signals from the ethylene and jasmonate pathways in activating defense-related genes for necrotrophic pathogens [50–51].

The association on chromosome 6 contained three candidate genes related to plant defense, which encoded phosphatidylinositol kinase (*GRMZM2G132373*), phosphogluconate dehydrogenase (*GRMZM2G431708*) and beta-glucosidase (*GRMZM2G055699*), respectively. Phosphatidylinositol kinase is a crucial component of many signaling pathways by acting through localized modulation of phosphatidylinositol 3-phosphate levels, which is important for *phytophthora* pathogens infection in plant [52]. Phosphogluconate dehydrogenase is involved in the generation of reactive oxygen species (ROS), the first events after elicitation of the hypersensitive response (HR), and plant defense could benefit from improved NADPH availability due to increased phosphogluconate dehydrogenase activity in the cytosol [53]. Beta-glucosidase is a bio-activating component, and can catalyze phytoanticipins, a type of secondary metabolites against pathogens in plant defense [54].

It was reported repeatedly there was a major QTL on chromosome 8 (bin 8.03) for resistance to MRDD in maize across different mapping populations [9, 26], and the detailed study showed that the major QTL (*qMrdd1*) was delimited to a region of 1.2 Mb [26]. Within *qMrdd1* region, we identified a candidate gene (*GRMZM2G110739*) encoding Mlo-like protein. Interestingly, both the *qMrdd1* and *mlo* are recessively inherited, and the recessive *mlo* mutation in barley confers broad-spectrum resistance to biotrophic *Blumeria graminis f*. *sp*. *hordei*, the causal pathogen of powdery mildew disease [55–56]. In an independent study of gene expression profile in RBSDV-infected maize, *mlo* homologue gene was altered dramatically with MRDD symptom development [58], which implied the importance of *mlo* in resistance to MRDD in maize.

In present study, combined analysis of 17 variants with resistance to MRDD was conducted by using linear regression model, where we summed the number of resistance alleles carried by each individual. This assumed that each of the alleles had the similar additive effect on resistance to MRDD. Within the current association mapping population, we found strong and negative correlation between the MRDD severity and number of resistance alleles, and the lines carrying more resistance alleles tended to have a higher resistance to MRDD. It implied there may have multiple genes to affect MRDD resistance in maize, genome wide selection should be a good alternative for resistance breeding. Interestingly, we found some highly resistant lines in tropic germplasm and GEM project having relatively small number of resistance alleles. As shown in S2 Fig, four resistant lines (i.e., CML115, CIMBL146, CIMBL39 and GEMS11) were identified with resistance alleles number ranged from 3 to 5 and diverged from the regression curve significantly. The exception may be due to the existence of rare resistance allele which could not identified by GWAS in present association mapping population. It is a common phenomenon that allele frequencies are significantly different for some important genes in tropical and temperate germplasm, and the clear examples are the pro-vitamin A gene *lyce1* [59] and *crtRb1* [60]. In light of the severity of MRDD in temperate maize breeding program, it is worth developing linkage mapping population to detect the new alleles or haplotypes. The newly identified resistant lines with tropical background should be considered in the future breeding program.

In present study, we need to concern that all candidate gene information is obtained based on the reference genome B73 information which is a susceptible line to most diseases. The resistance alleles may have been fully deleted in susceptible line as a recent report case in maize [61]. Therefore, validation of the candidate genes identified by GWAS in present study is necessary in additional studies before any application. For example we can scan the BAC library of resistant line with linked markers and look for the possible resistance genes and alleles then validate them with additional approaches (e.g., transgenic or/and validate in near isogenic line population).

## Supporting Information

S1 Fig The phenotypic distribution of resistance to MRDD in association panel. Phenotypic data were the BLUP values across environments (i.e., the phenotypic data from 2012 and 2013 together with the extremely resistant lines in 2011).
(TIF)

S2 Fig The correlation of the number of resistance alleles in each line with resistance to MRDD in association panel. The dots with black color stand for the lines derived from US hybrid P78599, and the dots with red color stand for the resistant lines with low number of resistance alleles. The numbers from 1 to 13 stand for JH59, DAN3130, DH29, ZHONG69, 18–599, P138, DAN599, P178, QI319, GEMS11, CIMBL146, CIMBL39 and CML115, respectively.
(TIF)

S1 Table The list of the lines and their phenotypic evaluation to MRDD in association panel across environments. The phenotypic data (DSI) were the BLUP values across environments (i.e., the phenotypic data from 2012 and 2013 together with the extremely resistant lines in 2011).
(PDF)
