# Supplementary figures and images for: Genome-Wide Association Implicates Candidate Genes Conferring Resistance to Maize Rough Dwarf Disease in Maize
Source: PLoS One. 2015 Nov 3;10(11):e0142001. doi: 10.1371/journal.pone.0142001 (PMC4631334; doi:10.1371/journal.pone.0142001)

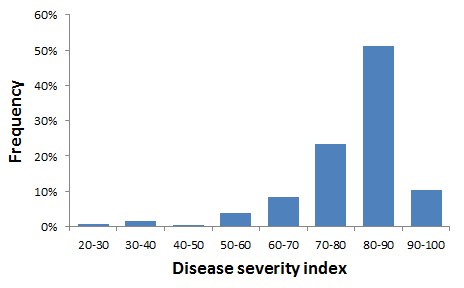

Supplement: S1 Fig — Phenotypic data were the BLUP values across environments (i.e., the phenotypic data from 2012 and 2013 together with the extremely resistant lines in 2011). (TIF) [file pone.0142001.s001.tif]

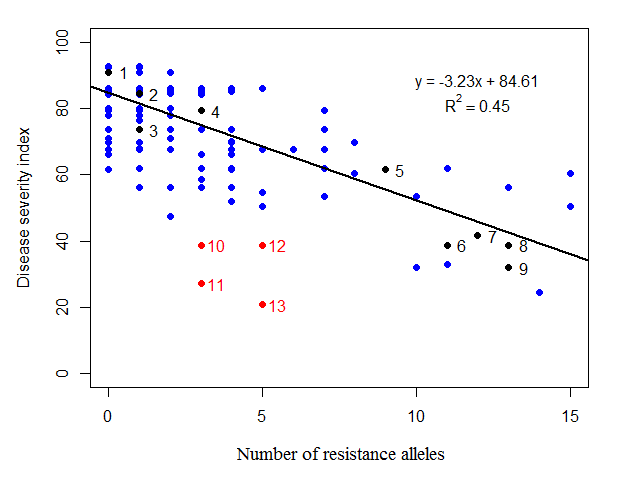

Supplement: S2 Fig — The dots with black color stand for the lines derived from US hybrid P78599, and the dots with red color stand for the resistant lines with low number of resistance alleles. The numbers from 1 to 13 stand for JH59, DAN3130, DH29, ZHONG69, 18–599, P138, DAN599, P178, QI319, GEMS11, CIMBL146, CIMBL39 and CML115, respectively. (TIF) [file pone.0142001.s002.tif]
