# Supplementary material for: Genome-Wide Association Implicates Candidate Genes Conferring Resistance to Maize Rough Dwarf Disease in Maize
Source: PLoS One. 2015 Nov 3;10(11):e0142001. doi: 10.1371/journal.pone.0142001 (PMC4631334; doi:10.1371/journal.pone.0142001)
Supplement: S1 Table — The phenotypic data (DSI) were the BLUP values across environments (i.e., the phenotypic data from 2012 and 2013 together with the extremely resistant lines in 2011). (PDF) [file pone.0142001.s003.pdf]

S1 Table. The list of the lines and their phenotypic evaluation to MRDD in the association panel across environments.

**Lines**	**DSI**	**Pedigree**	**Origin**
150	86.0	Unkown	China
177	73.6	Unkown	China
238	86.0	Unkown	China
268	73.6	(shen5003×U8112) F3×32	China
501	33.0	Unknown	China
647	85.3	Landrace	China
812	86.0	Improved from U8112	China
1323	53.4	Unknown	China
1462	85.1	Unkown	China
3411	86.0	Unknown	China
4019	62.0	G108×G172	China
5213	86.0	Improved from Mo17	China
5237	79.8	Dan340×HuangZaoSi	China
5311	85.1	GuiZhouQingLongWuSuiBai	China
7327	84.8	S37×3H-2	China
7381	60.4	Unknown	China
8902	84.4	Ye107×81162	China
9642	73.6	Unkown	China
9782	85.1	American Hybrid	China
81162	90.9	(AiJin525×Ye107)×106	China
526018	73.6	Unkown	China
04K5686	86.0	Landrace	China
05W002	84.4	05W002-1	China
05WN230	92.2	05WN230-1	China
07KS4	86.0	Unknown	China
18-599	61.5	American Hybrid P78599	China
303WX	86.0	Landrace	China
384-2	32.0	Unknown	China
3H-2	85.3	(WeiDa202×Zi330)×H84	China
4F1	73.6	Improved from Mo17 by radiation	China
7884-4Ht	85.1	78-6×H84	China
835a	56.2	U8112×Ye515	China
835b	79.4	U8112×Ye515	China
B11	86.0	Landrace	China

B110	79.8	BS13(S)C5	China
B111	86.0	BSSS(R)C9	China
B113	86.0	BS11(FR)C9	China
B151	86.0	Unkown	China
B73	85.1	BSSS	China
B77	86.0	B77-3	China
BEM	79.8	Landrace	China
BS16	86.0	Population BS16	China
By4839	79.4	BHO	China
By4944	86.0	BHO	China
By4960	85.1	BHO	China
By804	85.1	BHO	China
By807	86.0	BHO	China
By809	86.0	BHO	China
By813	85.1	BHO	China
By815	85.1	BHO	China
By843	85.1	BHO	China
By855	73.6	BHO	China
BZN	79.8	Landrace	China
C8605	90.9	Tie7922×Shen5003	China
CF3	86.0	Unknown	China
Chang7-2	85.1	HuangZaoSi×WeiChun	China
Cheng698	73.6	Foreign Hybrid	China
D863F	24.5	Unkown	China
Dan3130	84.4	American Hybrid P78599	China
Dan340	79.8	Lv9×Wide Pod Corn	China
Dan360	86.0	Lv9×Wide Pod Corn	China
Dan4245	84.4	Dan4245-1-3-1-3-1-1	China
Dan598	67.8	(Dan340×Danhuang11)×(Danhuang02×Dan599)	China
Dan599	41.7	American Hybrid P78599	China
DH29	73.6	American Hybrid P78599	China
DH3732	85.1	DH3732-1-1-2-2-1-1	China
Dong237	85.1	Synthetic	China
Dong46	85.1	M14	China
EN25	86.0	Unkown	China
ES40	90.9	Landrace	China
FCD0602	69.7	Unkown	China

Gy1032	79.8	AIHO	China
Gy220	79.4	AIHO	China
Gy237	90.9	AIHO	China
Gy386	73.6	AIHO	China
GY386B	86.0	GY386-1	China
Gy462	85.1	AIHO	China
Gy798	86.0	AIHO	China
HSBN	85.1	Landrace	China
HTH-17	92.5	HuoTangBai42×Hai1917× Mo17Ht	China
Hu803	85.3	U8112×Shen5003	China
HuangC	86.0	(HuangXiao162×Zi330/O2)×Tuxpenno	China
HYS	69.7	(HuangZaoSi×YeJiHong)×HuangZaoSi	China
HZS	90.9	TangSiPingTou	China
IRF291	85.3	A671×LH38	China
IRF314	85.1	(PA91/LH98A)-6-420-64-2-1-3-1-1-1	China
J4112	86.0	A619×U8112	China
JH59	90.9	American Hybrid P78599	China
JH96C	85.1	Synthetic	China
Ji53	79.4	Derived from Ji Synthetic C0-2	China
Ji63	84.1	(127-32×Tie84)×(Wei24×Wei20)	China
Ji842	86.0	Ji63×Mo17	China
Ji846	86.0	Ji63×Mo17	China
Ji853	73.6	(HuangZaoSi×Zi330)×Zi330	China
Jiao51	86.0	Improved from Landrace	China
JY01	73.6	JiYan01-3-2-2-1-5-1	China
K10	90.9	(Chang3×Shen5003)×Chang3	China
K12	85.1	HuangZaoSi×WeiChun	China
K22	79.4	K11×Ye478	China
L3180	85.1	Foreign hybrid	China
LG001	86.0	Unknown	China
Liao138	85.3	Improved from Dan340	China
Liao159	86.0	Unkown	China
Liao5114	85.1	Tie7922×Shen5003	China
Liao5262	86.0	Liao526-2-2-2-1-2-1	China
Liao5263	85.1	Liao526-2-2-2-1-3-1	China
LK11	85.1	Mo17×Zi330	China
Lv28	85.1	LvDaHongGu	China

Lx9801	92.5	Xi502×H21	China
LXN	86.0	Landrace	China
LY	79.4	Unknown	China
LY042	68.1	Unkown	China
M153	85.1	Unknown	China
M165	85.1	Unknown	China
M97	79.8	Unknown	China
MO113	85.3	Unknown	China
Mo17	86.0	187-2×C103	China
Nan21-3	79.8	Jugoslavian Hybrid	China
P138	38.8	American Hybrid P78599	China
P178	38.8	American Hybrid P78599	China
Q1261	67.8	Improved from K12	China
Qi319	31.9	American Hybrid P78599	China
R08	85.1	American Hybrid P78641	China
R15	79.8	American Hybrid	China
R15X1141	85.3	R15*X1141P-3-3-3-1-1-2	China
Ry684	85.1	RYD	China
Ry697	79.8	RYD	China
Ry713	86.0	RYD	China
Ry729	85.1	RYD	China
Ry737	85.3	RYD	China
S22	92.2	American Hybrid	China
Shen137	50.4	American Hybrid 6JK611	China
Shen5003	85.3	American Single-cross 3147	China
Si273	85.1	American Hybrid	China
Si434	86.0	Si466×Hua94	China
Si444	85.1	A619×HuangZaoSi	China
SW92E114	86.0	Landrace	China
Sy1032	86.0	Syn.D.O	China
Sy1035	84.4	Syn.D.O	China
Sy1039	92.5	Syn.D.O	China
Sy1052	86.0	Syn.D.O	China
Sy3073	85.1	Syn.D.O	China
Sy999	85.1	Syn.D.O	China
Tian77	85.1	(HuangXiao162×Mei1865)×(187-2×Nan55)	China
Tie7922	79.8	American Hybrid 3382	China

TT16	62.0	TianTai16-1-3-1-2-2-2	China
TY1	56.2	TY30331-2-4-2-1-3-2	China
TY10	85.1	TY30331-2-8-1-1-3-2	China
TY11	85.1	TY30331-2-8-1-2-1-1	China
TY3	85.1	TY30331-2-4-2-2-2-1	China
TY4	86.0	TY30331-2-4-2-2-2-2	China
TY5	56.2	TY30331-2-4-2-2-3-1	China
TY6	85.1	TY30331-2-5-1-2-3-2	China
TY7	56.2	TY30331-2-5-2-1-1-3	China
TY8	85.1	TY30331-2-7-1-2-1-1	China
TY9	85.1	TY30331-2-8-1-1-3-1	China
U8112	85.3	American Hybrid 3382	China
W138	90.9	Unknown	China
WH413	90.9	HuangZaoSi×WenQing1331	China
Wu109	86.0	XiDan7	China
Xi502	84.1	Dan340×HuangZaoSi	China
Xun971	79.8	Landrace	China
XZ698	86.0	XinZhong698-3-1-1-2-1-2-3	China
Ye107	90.9	Foreign Hybrid XL80	China
Ye515	86.0	(HuaFeng100×AiGo352)×HuangZaoSi	China
Ye52106	86.0	(Ye1075×Ye106)×AiJin525	China
Ye8001	79.8	Ye488×3189	China
Yu374	79.8	Synthetic	China
Yu87-1	56.2	American Hybrid	China
Z2018F	85.1	Zheng2018F2*698-3-1-2-1-1	China
ZaC546	86.0	Variant plants from C103	China
ZB648	69.7	ZhongBei648-1	China
ZH68	-	American Hybrid P78599	China
Zheng28	69.7	Ye478×Tuxpeno	China
Zheng29	85.1	Shen5003	China
Zheng30	79.4	Zheng20×Ye478	China
Zheng32	85.3	American Single-cross 3382	China
Zheng35	86.0	Unkown	China
Zheng58	86.0	Variant plants from Ye478	China
Zheng653	84.8	(Shen5003×Zong31)×Shen5003	China
Zhi41	90.9	Unkown	China
Zhong69	79.4	American Hybrid P78599	China

Zong31	85.1	Synthetic	China
ZZ01	67.8	American Hybrid P78641	China
ZZ03	67.8	Unkown	China
Gy923	-	AIHO	China
Gy1007	-	AIHO	China
Ry732	-	RYD	China
Sy998	-	Syn.D.O	China
Sy1077	-	Syn.D.O	China
Sy1128	-	Syn.D.O	China
Ye478	-	U8112×Shen5003	China
Zi330	-	OH43×KeLi67	China
Zheng22	-	Dan340×E28	China
chuan48-2	-	Synthetic	China
Si446	-	Qu43×Zi330	China
Chang3	-	Improved from Landrace	China
H21	-	HuangZaoSi×H84	China
832	-	American Single-cross	China
Ye488	-	U8112×Shen5003	China
BT1	-	8085×Thai Hybrid	China
Dan9046	-	Shen5003×Tie7922	China
CY72	-	Improved from Landrace	China
Hai268	-	Improved from Landrace	China
Hai1134	-	HuangYa×Hua94	China
SW1611	-	Suwan2	China
Zong3	-	Synthetic	China
Yan414	-	HuangZaoSi	China
Qi205	-	(VeiAi141×ZhongXi017)×Population 70	China
S37	-	Suwan1	China
K14	-	Shen5005×6917	China
TX5	-	Landrace	China
BGY	-	Landrace	China
HB	-	Huojiabaimaya	China
DSB	-	Landrace	China
WMR	-	Landrace	China
MN	-	Landrace	China
Hua83-2	-	Landrace	China
NMJT	-	Landrace	China

04K5702	-	Landrace	China
04K5672	-	Landrace	China
D047	-	Landrace	China
QTHHSBTS	-	Landrace	China
CA47	-	pool 33	China
X1141P	-	X1141P-1-4-6-1-3-2-1-1	China
975-12	-	78698×Dan9046	China
NX15	-	Landrace	China
YZ15	-	YuZong15BC-2	China
TY2	-	TY30331-2-4-2-2-1-1	China
CIMBL10	73.6	(((CML 408 / B104) x (CML 411/ B104))-1-1-BB)-B	CIMMYT
CIMBL100	79.8	(LAMA2002-43-2-B)-B	CIMMYT
CIMBL101	73.6	(LAMA2002-46-2-B)-B	CIMMYT
CIMBL102	67.8	(LAMA2002-53-5-B)-B	CIMMYT
CIMBL105	86.0	(LAMA2002-61-1-B)-B	CIMMYT
CIMBL106	85.3	(MAS[206/312]-23-2-1-1-BBB/[BETASYN]BC1-10-2-1-#-B)-B	CIMMYT
CIMBL107	86.0	(MAS[206/312]-23-2-1-1-BBB/[BETASYN]BC1-11-3-1-1-1)-B	CIMMYT
CIMBL108	77.9	(MAS[206/312]-23-2-1-1-BBB/[BETASYN]BC1-3-7-1-1-1)-B	CIMMYT
CIMBL109	73.6	(MAS[206/312]-23-2-1-1-BBB/[BETASYN]BC1-6-1-1-1-1)-B	CIMMYT
CIMBL11	86.0	(((CML150xCML451)-B-33-3-1-BBx(CML176 x CL-G2501)-B-43-1)-B-38-1-2-1-1-B)-B	CIMMYT
CIMBL110	86.0	(MAS[206/312]-23-2-1-1-BBB/[BETASYN]BC1-9-3-1-1-1)-B	CIMMYT
CIMBL111	86.0	(MAS[MSR/312]-117-2-2-1-BBB/[BETASYN]BC1-11-5-2-1-1)-B	CIMMYT
CIMBL113	86.0	(MIRTC4Am F17-B-2-1-BB)-B	CIMMYT
CIMBL114	84.8	(Nei 402025-BBB)-B	CIMMYT
CIMBL115	85.3	(Nei 9008-BBB)-B	CIMMYT
CIMBL116	84.1	(OBATANPA-SRc1F3(balbulk1)-#bal/[BETASYN]BC1-54-1-2-BBB)-B	CIMMYT
CIMBL117	86.0	(OBATANPA-SRc1F3(balbulk1)-#bal/[BETASYN]BC1-65-2-1-BB)-B	CIMMYT
CIMBL119	86.0	(P390Am/CMLc4F253-B-2-2-4-1-BB)-B	CIMMYT
CIMBL12	92.5	(((CML150xCML451)-B-33-3-1-BBx(CML176 x CL-G2501)-B-43-1)-B-38-1-2-2-1-B)-B	CIMMYT

CIMBL120	85.1	(P391c2 F22-1-1-2-1-BBB)-B	CIMMYT
CIMBL122	86.0	(P591c4 1 y 2 GEN F3-1-1-2-B-B-B)-B	CIMMYT
CIMBL124	92.2	(P84c3BcxLine recycle LL TpreAsiaxMIRT F59-2-1-1-1-BB)-B	CIMMYT
CIMBL125	69.7	(P84c3BcxLLTtardAsiaxMIRTF41-2-1-2-2-BB)-B	CIMMYT
CIMBL126	73.6	(Pob . 391 C4 F91-1-2-1-BB)-B	CIMMYT
CIMBL127	70.0	(Pop.147-F2#132-1-1-B-2-BBBB)-B	CIMMYT
CIMBL129	79.4	(pop.69 Templado Amarillo QPM -BBB1-6-BBB)-B	CIMMYT
CIMBL133	86.0	(SAM4(Angola)/[BETASYN]BC1-66-1-3-BBB)-B	CIMMYT
CIMBL134	86.0	(SAM4(Angola)/[BETASYN]BC1-79-2-3-BB)-B	CIMMYT
CIMBL138	90.9	(TX03-1004-168-B)-B	CIMMYT
CIMBL139	67.8	(TX03-1004-179-B)-B	CIMMYT
CIMBL14	73.6	(((Ko326y x Tx806)-6-1-1-1-BB/CML161) x (Tx802/CML161))-1-BBB)-B	CIMMYT
CIMBL140	90.9	(TX03-1004-20-B)-B	CIMMYT
CIMBL141	67.8	(TX03-1004-73-B)-B	CIMMYT
CIMBL142	70.8	(TX03-1005-15-B)-B	CIMMYT
CIMBL143	90.9	(TX03-1006-22-B)-B	CIMMYT
CIMBL144	85.3	(TX03-1007-18-B)-B	CIMMYT
CIMBL145	84.4	(TX03-1007-36-B)-B	CIMMYT
CIMBL146	27.2	(TX03-1008-6-B)-B	CIMMYT
CIMBL147	85.1	(TX03-1013-186-B)-B	CIMMYT
CIMBL150	56.2	(TX03-1034-46-B)-B	CIMMYT
CIMBL152	77.9	(TX03-1035-55-B)-B	CIMMYT
CIMBL153	90.9	(TX03-1037-3-B)-B	CIMMYT
CIMBL154	73.6	(TZI18-BBBB)-B	CIMMYT
CIMBL155	86.0	(ZM305/[BETASYN]BC1-29-1-1-B)-B	CIMMYT
CIMBL157	84.4	(ZM305/[BETASYN]BC1-59-1-2-BBB)-B	CIMMYT
CIMBL17	85.1	((CML 326/B104)-B-9-BBBB)-B	CIMMYT
CIMBL18	85.1	((CML161 x CL-02603)-B-22-2-2-1-BBBB)-B	CIMMYT
CIMBL20	56.2	((CML161xCML413)-Bx(CML172 x CML451)-B-B-25-1-1-3-2-B)-B	CIMMYT
CIMBL21	47.5	((CML285/NC300)-B-6-BBBB)-B	CIMMYT
CIMBL22	73.6	((CML454 x CL-02603)-B-20-1-2-BB)-B	CIMMYT
CIMBL23	79.4	((CML454 x CL-02603)-B-7-2-2-BB)-B	CIMMYT
CIMBL25	77.9	((CML479/CLQ-RCYQ41)-B-2-1-2-2-1-B)-B	CIMMYT
CIMBL27	73.6	((CML479/CLQ-RCYQ41)-B-3-2-3-4-2-B)-B	CIMMYT
CIMBL28	85.1	((CML479/CLQ-RCYQ41)-B-53-1-1-2-1-B)-B	CIMMYT

CIMBL29	86.0	((CML481 x CML454)-B-8-1-2-BB)-B	CIMMYT
CIMBL30	67.8	((KU1409/DE3/KU1409)S2-18-2-BB)-B	CIMMYT
CIMBL32	56.2	((KU1409/KU1414-SR/KUI2007)-S2-2-1-BB)-B	CIMMYT
CIMBL33	62.0	((KU1409/KU1414-SR/KVI11)-S2-10-2-BB)-B	CIMMYT
CIMBL34	77.9	((KU1409/KU1414-SR/KVI3)-S2-3-2-BB)-B	CIMMYT
CIMBL38	56.2	((KU1409/KU1414-SR/SC213)-S2-10-1-BB)-B	CIMMYT
CIMBL39	38.8	((KU1409/KU1414-SR/SC55)-S2-12-1-BB)-B	CIMMYT
CIMBL40	86.0	((KU1409/SC55/KU1409)-S2-12-1-BB)-B	CIMMYT
CIMBL43	86.0	((P69Qc3HC107-1-1#-4-2#-4-B-B-1-4-B-B-B-B-BXCML 193)-B-B-2-B-B-B)-B	CIMMYT
CIMBL44	90.9	((SYN-Y-STR-34-1-1-1-1-2-1-BBBBB/NC354/SYN-Y-ST R-34-1-1-1-1-2-1-BBBBB)-S2-13-1-BB)-B	CIMMYT
CIMBL45	86.0	((Tx601XB104-B/B110XFR2128-B)-BB-4-BBB)-B	CIMMYT
CIMBL46	90.9	([[[NAW5867/P30SR]-43-2/[NAW5867/P30SR]-114-1]-9-3-3-B-1-B/CML395-1]-B-13-1-B-4-#/[BETASYN]BC1-8-1-1-1-1)-B	CIMMYT
CIMBL47	85.3	([DTPYC9-F11-2-3-1-1-BB x DTPYC9-F46-1-2-1-1-B]-B-1-1-B)-B	CIMMYT
CIMBL48	85.1	([DTPYC9-F46-1-2-1-1-B x DTPYC9-F74-1-1-1-1-BB]-B-4-1-B)-B	CIMMYT
CIMBL49	90.9	([DTPYC9-F65-2-2-1-1-BB x DTPYC9-F65-2-3-1-1-BB]-2-2-2-B)-B	CIMMYT
CIMBL50	84.8	([DTPYC9-F74-1-1-1-1-BB x DTPYC9-F65-2-2-1-1-BB]-B-3-2-B)-B	CIMMYT
CIMBL51	77.9	([GQL5/[GQL5/[MSRXPOOL9]C1F2-205-1(OSU23i)-5-3-X-X-1-B-B]F2-4sx]-8-6-BB/[BETASYN]BC1-6-5-1-1-1)-B	CIMMYT
CIMBL52	79.8	([GQL5/[GQL5/CML202]F2-1sx]-3-1-2-B/[BETASYN]BC1-2-5-1-1-1)-B	CIMMYT
CIMBL53	69.7	([SAM4/BETASYN]BC2FS1-1-1-1-BB)-B	CIMMYT
CIMBL54	84.4	([SAM4/BETASYN]BC2FS21-4-3-B)-B	CIMMYT
CIMBL55	86.0	([SAM4/BETASYN]BC2FS36-4-1-2-BB)-B	CIMMYT
CIMBL56	85.3	([SAM4/BETASYN]BC2FS9-2-2-1-BB)-B	CIMMYT
CIMBL57	86.0	([ZM305/BETASYN]BC2-133-1-1-BB)-B	CIMMYT
CIMBL58	73.6	([ZM305/BETASYN]BC2-133-1-2-BBB)-B	CIMMYT
CIMBL59	61.5	([ZM305/BETASYN]BC2-182-1-2-BB)-B	CIMMYT
CIMBL6	90.9	(Ac8730SR-##-124-1-5-B-1-#/[BETASYN]BC1-16-2-3-1-1)-B	CIMMYT
CIMBL60	73.6	([ZM305/BETASYN]BC2-273-1-1-BB)-B	CIMMYT
CIMBL61	85.1	(Carotenoid Syn3-FS11-4-3-BBB)-B	CIMMYT

CIMBL62	92.2	(Carotenoid Syn3-FS4-2-4-BBB)-B	CIMMYT
CIMBL63	79.8	(Carotenoid Syn3-FS5-1-5-BB)-B	CIMMYT
CIMBL66	86.0	(CL-G1839 G18seqC3-17-1-1-2-2-B*5)-B	CIMMYT
CIMBL67	77.9	(CL-G2606 G26SeqC1-149-1-1-2-1-2-1-BB)-B	CIMMYT
CIMBL68	67.8	(CL-G2609 G26C23-75-1-1-2-1-B*5)-B	CIMMYT
CIMBL69	66.2	(CL-G2609-B)-B	CIMMYT
CIMBL7	86.0	(AMATLC0HS71-1-1-2-1-1-1-BBBBBBB)-B	CIMMYT
CIMBL70	90.9	(CL-G2611 G26SEQF135-3-3-1-1-1-2-BB)-B	CIMMYT
CIMBL71	86.0	(CL-RCY007=PIO3011F2-B)-B	CIMMYT
CIMBL73	86.0	(CML329/MBR C3 AmF25-2-1-1-BBB)-B	CIMMYT
CIMBL74	85.1	(CML445/[BETASYN]BC1-2-2-5-1-1)-B	CIMMYT
CIMBL75	85.1	(CML445/[BETASYN]BC1-9-2-3-1-1)-B	CIMMYT
CIMBL76	90.9	(CML488/[BETASYN]BC1-8-6-1-1-1)-B	CIMMYT
CIMBL77	76.2	(CML489/[BETASYN]BC1-2-#-B)-B	CIMMYT
CIMBL78	85.3	(CML489/[BETASYN]BC1-5-2-1-2-1)-B	CIMMYT
CIMBL79	84.8	(CML489/[BETASYN]BC1-7-2-1-1-1)-B	CIMMYT
CIMBL8	73.6	(((B104/NC300)x(CML-415/B104))-4-2-BB)-B	CIMMYT
CIMBL81	69.7	(Cuba / Guad C3 F125 -2-2-1-BBB)-B	CIMMYT
CIMBL82	84.4	(Cuba/Guad C3 F42-2-1-1-BBB)-B	CIMMYT
CIMBL83	90.9	(DRPYC9-F13-2-1-1-1-B)-B	CIMMYT
CIMBL84	86.0	(DTPYC9-74-1-1-1-1-B)-B	CIMMYT
CIMBL85	86.0	(DTPYC9-F11-2-3-1-2-B)-B	CIMMYT
CIMBL86	73.6	(DTPYC9-F116-2-1-1-1-B)-B	CIMMYT
CIMBL87	84.8	(DTPYC9-F125-2-8-1-1-B)-B	CIMMYT
CIMBL88	54.7	(DTPYC9-F134-3-1-B)-B	CIMMYT
CIMBL89	86.0	(DTPYC9-F143-5-4-1-2-B)-B	CIMMYT
CIMBL9	86.0	(((B104/NC300X(CML285/B104))-2-3-B-B)-B	CIMMYT
CIMBL90	90.9	(DTPYC9-F38-4-6-1-1-B)-B	CIMMYT
CIMBL91	86.0	(DTPYC9-F46-1-7-1-1-BBBB)-B	CIMMYT
CIMBL92	86.0	(DTPYC9-F46-3-9-1-2-B)-B	CIMMYT
CIMBL93	86.0	(DTPYC9-F65-2-3-1-1-BBBBB)-B	CIMMYT
CIMBL94	77.9	(DTPYC9-F69-3-1-1-1-BBBBB)-B	CIMMYT
CIMBL95	62.0	(Florida A plus Syn-FS2-2-1-BB)-B	CIMMYT
CIMBL96	86.0	(KUI carotenoid syn-FS11-1-1-BBB)-B	CIMMYT
CIMBL97	79.4	(KUI carotenoid syn-FS17-3-1-BBB)-B	CIMMYT
CIMBL99	52.0	(LAMA2002-10-1-B)-B	CIMMYT
CML115	20.7	P45C2F151(RIF)-#-5-1-2-BBB	CIMMYT

CML116	73.6	P45(STE)C1F36-5-1-3-1-BB	CIMMYT
CML118	86.0	SIYFS3#B1-7-B1-B1#B1	CIMMYT
CML122	67.8	(MP704/MP78:518)-8-3-4-B1-4-2-3-B1-B1#B1	CIMMYT
CML130	86.0	P33C1F118-5-1-4-5-4-B1-B1#B1	CIMMYT
CML134	67.8	P45C2F151-1-2-5-3-1-2-4-B1-B1#B1	CIMMYT
CML139	86.0	MP78:518-15-B1-B1#B1	CIMMYT
CML162	79.8	G25QC1F18-8-1-2-BB-2-BBB	CIMMYT
CML163	86.0	G26QH31-2-2-#-2-2-1-B-1-BB-#	CIMMYT
CML165	85.1	P66C1F144-3-1-1-BB-1-BB-#	CIMMYT
CML170	69.7	G26QC22H9-3-1-5-1-BB	CIMMYT
CML171	85.1	G25QS4B-H13-5-B-1-1-2-B-1-BBB	CIMMYT
CML172	85.1	G25QS4B-H35-2-B-1-1-2-B-4-BBBB	CIMMYT
CML191	86.0	G34QH146-1-1-4-B	CIMMYT
CML192	86.0	G34QH174-3-1-2-BB	CIMMYT
CML20	86.0	P24F34-2-3-B-###	CIMMYT
CML223	79.8	EV88SUWAN1SR(BC5)-3-1-2-BB	CIMMYT
CML225	79.8	EV88SUWAN1SR(BC5)-62-1-1-BB	CIMMYT
CML226	50.4	EV88SUWAN1SR(BC5)-78-1-1-BB	CIMMYT
CML228	85.3	EV88SUWAN1SR(BC5)-201-1-1-BB	CIMMYT
CML287	66.2	(P24F26/27F1)-4-1-B-1-1-BB-F	CIMMYT
CML290	86.0	(P28/TSR)-33-3-7-3-1-BB-F	CIMMYT
CML300	86.0	SINTAMTSR-76-1-2-3-1-BB-F	CIMMYT
CML304	60.4	SINTAMTSR-76-1-2-3-2-BB-F	CIMMYT
CML305	85.3	SINTAMTSR-23-2-2-1-3-BB-F	CIMMYT
CML31	86.0	P27F117-1-4-B-####	CIMMYT
CML32	61.5	P28(TSR)S2-11-2-4-B-1-##	CIMMYT
CML323	58.5	P33C2(STE)-102-2-B-2-B-1-B*3	CIMMYT
CML324	84.4	P33C2(STE)-12-1-B-2-B*4	CIMMYT
CML325	62.0	P45C6F28-1-2-B*6	CIMMYT
CML327	77.9	P45C6F83-3-1-B*5	CIMMYT
CML338	70.8	P590BF84-3-3-5-3-1-1-B-#-B	CIMMYT
CML360	86.0	SA4C2F(21/26)-1-2-2-2-B	CIMMYT
CML364	77.9	SAF1-5-1-1-5-3-B	CIMMYT
CML408	73.6	P24STEC2-29-BBBB-#-4-BBBBBBB	CIMMYT
CML411	77.9	P28C7-S4-#-BBBBBBBBBBB	CIMMYT
CML415	86.0	KC3001/KC3002-22-2-1-B-4-BBBBBBBB	CIMMYT
CML422	66.2	G17TSRH5-2-4-7-1-1-1-BBBBBBB	CIMMYT

CML423	90.9	G18C19H100#-4-1-1-BBBBBBB	CIMMYT
CML426	85.1	P31C4S5B-38-##-2-BBB	CIMMYT
CML431	85.3	AMATLC0HS170-2-3-2-1-1-1-1-B*3	CIMMYT
CML432	84.1	KTX3752F2-7-1-1-1-BBB	CIMMYT
CML433	85.1	KTX3753F2-5-1-1-2-BBB	CIMMYT
CML451	73.6	[NPH28-1/G25)/NPH28]-1-2-1-1-3-1-B*6	CIMMYT
CML454	86.0	CAPITANMIRANDA8627-20-1-2-B*15	CIMMYT
CML470	84.8	P31C4S5B-39-##-1-BB	CIMMYT
CML473	85.3	P31C4S5B-23-##-4-BBBB	CIMMYT
CML479	86.0	(P24STE-5/24STE-17)-BBBB-###-B-3-B-1-B*4	CIMMYT
CML480	77.9	SINTAMTSRC2-88-2-2-B*8	CIMMYT
CML486	53.4	P45C8-76-1-2-1-2-BBBB	CIMMYT
CML493	77.9	P66C1F22-2-5-2-BBBB-3-BB	CIMMYT
CML496	69.7	P36C9F90-B-5-B*7	CIMMYT
CML50	85.1	AC8078-2-4-1-##	CIMMYT
CML51	73.6	STAROSA8079-1-2-3-###	CIMMYT
CML69	69.7	P36C5F37-2-1-1-B1#B1-B1#B1-B1	CIMMYT
CML114	-	P45C2F-B-88-###1-1-2-BB	CIMMYT
CML121	-	(PI218191/PI209135//PI226685/P1317328/3/P47/MPSWCB4) 6-3-1-5-1-B1-B1#B1(MP704/MP78:518)-8-3-4-B1-4-2-3-B1-B1-#B1	CIMMYT
CML166	-	P66C1F215-4-1-2-BB-2-BBB	CIMMYT
CML169	-	G26QC22H7-1-1-1-1-BB	CIMMYT
CML189	-	G34QH17-2-1-1-B	CIMMYT
CML229	-	EV88SUWAN1SR(BC5)-293-1-1-BB	CIMMYT
CML26	-	P26F40-1-4-1-3-#-2-B-##	CIMMYT
CML27	-	P27F1-1-3-B-###	CIMMYT
CML28	-	P27F35-8-3-B-####-B	CIMMYT
CML282	-	(G22F128/G22F25)-2-2-3-1-BB-F	CIMMYT
CML285	-	P24C5F34-2-3-B-F-2#-BBB-F	CIMMYT
CML289	-	P24STEC1F23-5-2-1-2-3-BB-F	CIMMYT
CML29	-	P27F71-3-1-B-#*5-B	CIMMYT
CML298	-	SINTAMTSR-7-4-2-2-1-BB-F	CIMMYT
CML307	-	SINTAMTSR-61-3-2-8-2-BB-F	CIMMYT
CML326	-	P45C6F6-2-1-1-B*5	CIMMYT
CML361	-	SA4C2F(21/26)-4-2-7-3-B	CIMMYT
CML40	-	P36C5F144-2-2-B-###	CIMMYT

CML412	-	P36C5F279-1-1-B-F-##-B	CIMMYT
CML428	-	SW91145-2P3S2-##-3-BBBB	CIMMYT
CML430	-	AMATLC0HS169-1-1-1-1-2-2-1-B*3	CIMMYT
CML465	-	AMATLC0HS71-1-1-2-1-1-1-BBB	CIMMYT
CML468	-	P28C9F113-3-1-4-B*7	CIMMYT
CML474	-	SW92145-2EV-13-1-BB	CIMMYT
CML497	-	[CL00331*v]-3-B-3-2-1-B*5	CIMMYT
CML113	-	P33C1F-B-129-B-###-2-B	CIMMYT
CML168	-	G26QSINT-31-1-2-2-BB	CIMMYT
CML297	-	SINTAMTSR-23-3-1-1-1-BB-F	CIMMYT
CIMBL13	-	(((CML285/B104)x(CML288/NC300))-2-1-BB)-B	CIMMYT
CIMBL15	-	((CL-RCY020 x CL-02450)-B-40-1-1-BB)-B	CIMMYT
CIMBL16	-	((CML 288/NC300)-B-9-B-1-BBB)-B	CIMMYT
CIMBL19	-	((CML161xCML413)-Bx(CML172 x CML451)-BB-25-1-1-2-2-B)-B	CIMMYT
CIMBL24	-	((CML454 x CML451)-B-7-3-2-BB)-B	CIMMYT
CIMBL26	-	((CML479/CLQ-RCYQ41)-B-2-1-2-2-2-B)-B	CIMMYT
CIMBL31	-	((KU1409/KU1414-SR/CI187)-S2-10-2-BB)-B	CIMMYT
CIMBL35	-	((KU1409/KU1414-SR/KVI43)-S2-4-2-BB)-B	CIMMYT
CIMBL36	-	((KU1409/KU1414-SR/M162W)-S2-3-1-BB)-B	CIMMYT
CIMBL37	-	((KU1409/KU1414-SR/NC350)-S2-1-2-BB)-B	CIMMYT
CIMBL41	-	((KU1414-SR/CML328/KU1414-SR)-S2 -12-1-BB)-B	CIMMYT
CIMBL42	-	((NC300/Tx772)-B-1-B2-BB)-B	CIMMYT
CIMBL64	-	(Carotenoid Syn3-FS8-4-1-BBB)-B	CIMMYT
CIMBL65	-	(CL-02841 Ac8928-40-1-1-1-1-B)-B	CIMMYT
CIMBL72	-	(CL-SCBY03)-B	CIMMYT
CIMBL80	-	(CTS013174/SW(S)C11-42-1-BB-1-3-BBBB/Nei9202-B)-B	CIMMYT
CIMBL98	-	(KUI carotenoid syn-FS25-3-2-BBB)-B	CIMMYT
CIMBL103	-	(LAMA2002-58-4-B)-B	CIMMYT
CIMBL104	-	(LAMA2002-60-9-B)-B	CIMMYT
CIMBL112	-	(MBR C6 AmF9-2-B-#-3-1-BBBBB)-B	CIMMYT
CIMBL118	-	(OBATANPA-SRc1F3(balbulk1)-#bal/[BETASYN]BC1-67-1-2-BBB)-B	CIMMYT
CIMBL121	-	(P591c4 1 y 2 GEN F205-1-1-1-BBB)-B	CIMMYT
CIMBL123	-	(P72c1xCML-297 x CL-02410-3-1-1-BBB)-B	CIMMYT
CIMBL128	-	(Pop.28C9HC113-3-1-4-B*-8-BBB)-B	CIMMYT
CIMBL130	-	(pop.70 Templado Amarillo QPM-BBB2-8-BBB)-B	CIMMYT

CIMBL131	-	(SAM4(Angola)/[BETASYN]BC1-43-1-1-B)-B	CIMMYT
CIMBL132	-	(SAM4(Angola)/[BETASYN]BC1-56-1-1-BBB)-B	CIMMYT
CIMBL135	-	(SAM4(Angola)/[BETASYN]BC1-82-1-2-BB)-B	CIMMYT
CIMBL156	-	(ZM305/[BETASYN]BC1-58-2-3-B)-B	CIMMYT
CIMBL2	73.6	Suwan 1	Thailand
CIMBL3	79.8	Suwan 1	Thailand
CIMBL4	79.8	Suwan 1 ; 2007 = DK version of Ki3	Thailand
A619	86.0	The University of Illinois, USA	USA
CI7	85.1	The University of Illinois, USA	USA
CIMBL1	79.4	derived from BSCB1(R)C9	USA
CIMBL5	-	H5*PX105A/H101	USA
DE.EX	84.4	The University of Illinois, USA	USA
GEMS1	86.0	PI503806xB94///B94	USA
GEMS10	79.4	2112-02_DK212T_S11_F2S4_9169-Blk20/00	USA
GEMS11	38.8	2116-02_DK212T_S11_F2S4_9172-Blk28/00	USA
GEMS12	85.1	2120-01_DK888_S11_F2S4_9175-Blk28/00	USA
GEMS13	73.6	2121-04_DK888_S11_F2S4_9178-Blk29/00	USA
GEMS14	73.6	2127-01_DK888_S11_F2S4_9181-Blk21/00	USA
GEMS15	77.9	2131-01_DK888_S11_F2S4_9184-Blk20/00	USA
GEMS16	90.9	2132-03_DK888_S11_F2S4_9187-Blk22/00	USA
GEMS17	79.4	2142-01_DK888_S11_F2S4_9190-Blk19/00	USA
GEMS18	85.1	2143-02_DK888_S11_F2S4_9193-Blk19/00	USA
GEMS19	90.9	2146-01_DK888_S11_F2S4_9196-Blk29/00	USA
GEMS2	90.9	FS8A(S):S09-43-2	USA
GEMS20	85.1	2150-01_DK888_S11_F2S4_9199-Blk16/00	USA
GEMS21	84.4	2152-02_DK888_S11_F2S4_65/97-Blk/97-99	USA
GEMS23	86.0	2156-02_DK888_S11_F2S4_h92847-Blk13/00	USA
GEMS24	67.8	2201-01_DK830_S11_F2S4_9208-Blk27/00	USA
GEMS25	86.0	2226-02_XL370A_S11_F2S4_9211-Blk25/00	USA
GEMS27	85.1	2250-01_XL370A_S11_F2S4_9214-Blk21/00	USA
GEMS28	86.0	2250-02_XL370A_S11_F2S4_3363-Blk03/00	USA
GEMS29	86.0	2253-01_XL370A_S11_F2S4_9220-Blk24/00	USA
GEMS3	79.8	2011-01_SE32-S17_F2S4_9148-Blk22/00	USA
GEMS30	90.9	2258-03_XL380_S11_F2S4_71/97-Blk/98	USA
GEMS31	90.9	2282-01_XL380_S11_F2S4_9226-Blk26/00	USA
GEMS32	86.0	2283-01_XL380_S11_F2S4_9229-Blk20/00	USA
GEMS33	86.0	PE001n16F2S2-44	USA

GEMS35	84.8	PE001n16F2S2-172	USA
GEMS36	77.9	PE001n16F2S2-176	USA
GEMS37	86.0	PE001n16F2S2-181	USA
GEMS39	73.6	PE001n16F2S2-239	USA
GEMS4	84.4	2084-02_DK212T_S11_F2S4_9151-Blk38/00	USA
GEMS40	73.6	PE001n16F2S2-431	USA
GEMS41	90.9	PE001n16F2S2-521	USA
GEMS42	86.0	PE001n16F2S2-705	USA
GEMS43	73.6	PE001n16F2S2-857	USA
GEMS44	77.9	CH05015:N12-140-1-B-B	USA
GEMS45	85.3	CH05015:N12-20-1-B-B	USA
GEMS46	85.1	CH05015:N15-8-1-B-B	USA
GEMS47	79.4	UR13085:N0125-014-001	USA
GEMS48	86.0	DKXL370:N11a20-31-1-B-B	USA
GEMS49	92.5	DKB844:S1601-512-1-B	USA
GEMS5	73.6	2086-01_DK212T_S11_F2S4_9154-Blk20/00	USA
GEMS50	85.3	DKB844:S1601-517-1-B	USA
GEMS51	73.6	CUBA164:S2008a-83-1-B	USA
GEMS52	90.9	CUBA164:S2008a-326-1-B	USA
GEMS53	90.9	CUBA164:S15-192-2-B	USA
GEMS54	79.8	ARI16035:S02-450-1-B	USA
GEMS55	85.3	ARI16035:S02-443-1-B-B	USA
GEMS57	79.8	ARI01150:N04-545-1-B	USA
GEMS58	79.8	ARI01150:N04-696-1-B	USA
GEMS59	86.0	SCROI:N1310-398-1-B	USA
GEMS6	90.9	2088-01_DK212T_S11_F2S4_9157-Blk29/00	USA
GEMS60	79.8	CHIS775:N1912-519-1-B-B	USA
GEMS61	90.9	ARI16026:S17-10-1-B-B	USA
GEMS62	79.8	ARI16026:S16-10-1-B-B	USA
GEMS63	86.0	CUBA164:S1511b-325-001	USA
GEMS64	85.1	CUBA164:S1511b-249-1-B-B	USA
GEMS65	90.9	CUBA117:S1520-41-1-B-B	USA
GEMS66	79.8	CUBA117:S1520-52-1-B-B	USA
GEMS9	85.1	2111-01_DK212T_S11_F2S4_9166-Blk31/00	USA
SC55	85.1	The University of Illinois, USA	USA
